# Safety and Tolerability of Subcutaneous IgPro20 at High Infusion Parameters in Patients with Primary Immunodeficiency: Findings from the Pump-Assisted Administration Cohorts of the HILO Study

**DOI:** 10.1007/s10875-020-00912-5

**Published:** 2021-01-06

**Authors:** John T. Anderson, Vincent R. Bonagura, Juthaporn Cowan, Connie Hsu, S. Shahzad Mustafa, Niraj C. Patel, John M. Routes, Panida Sriaroon, Donald C. Vinh, Jutta H. Hofmann, Michaela Praus, Mikhail A. Rojavin

**Affiliations:** 1 Clinical Research Center of Alabama, 504 Brookwood Blvd Suite 250, Birmingham, AL 35209 USA; 2 Donald and Barbara Zucker School of Medicine at Hofstra/Northwell, Great Neck, NY USA; 3 Hofstra-NS-LIJ School of Medicine, Feinstein Institute for Medical Research, Rm. 1236, 350 Community Drive, Manhasset, NY 11030 USA; 4 University of Ottawa, 501 Smyth Road, Box 223, Ottawa, ON K1H8L6 Canada; 5 Allergy & Immunology Specialists, PLLC, 13575 W. Indian School Road, Suite 200, Litchfield Park, AZ 85340 USA; 6 Rochester Regional Health, 222 Alexander Street, Suite 3000, Rochester, NY 14607 USA; 7 University of Rochester School of Medicine and Dentistry, 601 Elmwood Ave, Rochester, NY 14642 USA; 8 Department of Pediatrics, Levine Children’s Hospital, Atrium Health, 1000 Blythe Blvd, PO Box 32861, Charlotte, NC 28232 USA; 9 Department of Pediatrics, Children’s Hospital of Wisconsin, Medical College of Wisconsin, Medical College of Wisconsin, Milwaukee, 9000 W. Wisconsin Ave., Milwaukee, WI 53226 USA; 10 University of South Florida, 140 7th Ave. South, CRI 4008, St. Petersburg, FL 33701 USA; 11 McGill University Health Centre – Research Institute, 1001 Decarie Blvd, Block E, Rm EM3-3230 (Mail Drop: EM3-3211), Montreal, QC H4A 3J1 Canada; 12 CSL Behring AG, Wankdorfstrasse 10, 3014 Bern, Switzerland; 13 CSL Behring GmbH, Emil-von-Behring-Straße 76, 35041 Marburg, Germany; 14 CSL Behring LLC, 1020 First Avenue, King of Prussia, PA 19406 USA

**Keywords:** Primary immunodeficiency (PID), IgPro20, subcutaneous Ig (SCIG), pump-assisted infusion, high infusion volume, high infusion flow rate

## Abstract

**Purpose:**

To evaluate the safety and tolerability of subcutaneous IgPro20 (Hizentra^®^, CSL Behring, King of Prussia, PA, USA) administered at high infusion parameters (> 25 mL and > 25 mL/h per injection site) in patients with primary immunodeficiency.

**Methods:**

The Hizentra^®^ Label Optimization (HILO) study was an open-label, parallel-arm, non-randomized study (NCT03033745) of IgPro20 using a forced upward titration design for infusion parameters. Patients experienced with pump-assisted IgPro20 infusions received weekly IgPro20 infusions at a stable dose in the Pump-Assisted Volume Cohort (*N* = 15; 25–50 mL per injection site) and in the Pump-Assisted Flow Rate Cohort (*N* = 18; 25–100 mL/h per injection site). Responder rates (percentage of patients who successfully completed ≥ 75% of planned infusions), safety outcomes, and serum immunoglobulin G (IgG) trough levels were evaluated.

**Results:**

Responder rates were 86.7% (13/15, 25 mL) and 73.3% (11/15, 40 and 50 mL) in the Volume Cohort, and 77.8% (14/18, 25 and 50 mL/h), 66.7% (12/18, 75 mL/h), and 61.1% (11/18, 100 mL/h) in the Flow Rate Cohort. Infusion compliance was ≥ 90% in all patients in the Volume Cohort and in 83.3% of patients in the Flow Rate Cohort. The number of injection sites (Volume Cohort) and the infusion duration (Flow Rate Cohort) decreased with increasing infusion parameters. The rate of treatment-emergent adverse events per infusion was low (0.138 [Volume Cohort] and 0.216 [Flow Rate Cohort]). Serum IgG levels remained stable during the study.

**Conclusion:**

Pump-assisted IgPro20 infusions are feasible at 50 mL and 100 mL/h per injection site in treatment-experienced patients, which may result in fewer injection sites and shorter infusion times.

**Trial Registration:**

NCT03033745; registered January 27, 2017

**Supplementary Information:**

The online version contains supplementary material available at 10.1007/s10875-020-00912-5.

## Introduction

Primary immunodeficiency (PID) represents a heterogeneous group of disorders characterized by an intrinsic defect in one or more components of the immune system, such as antibody production. Patients with PID are more susceptible to recurrent infections, autoimmune diseases, and malignancies [1–3]. Immunoglobulin G (IgG) replacement therapy is effective in various types of cellular and antibody deficiencies to manage infections and other complications in these patients and can be administered intravenously or subcutaneously (SCIG) [4, 5].

IgPro20 (Hizentra^®^, CSL Behring, King of Prussia, PA, USA) is a ready-to-use formulation of polyvalent SCIG (highly purified IgG [≥ 98% purity]) with an IgG content of 20% [6]. It is approved for the treatment of PID in several countries including the USA, EU, Canada, Switzerland, Japan, and Australia [7, 8]. SCIG administration can be performed using an infusion pump or by manual push (also known as rapid push) using a syringe. Both infusion techniques have shown similar serum IgG levels for the same monthly dose and similar safety and tolerability profiles, although the comparative incidence of adverse events (AEs) varies between studies [9–12]. Therefore, the choice of SCIG administration technique can be tailored according to the individual patient’s preferences [13, 14].

At present, the maximal approved pump-assisted IgPro20 infusion parameters for PID in the USA are a volume of up to 25 mL per injection site and a flow rate of up to 25 mL/h per injection site [7]. Therefore, each pump-assisted infusion can take up to 2 h or more, depending on the dose, number of injection sites, and flow rate utilized [9, 15]. In the EU, pump-assisted IgPro20 infusions are approved at a volume up to 50 mL/site in adults [8]. EU recommendations for IgPro20 flow rates include initial pump-assisted infusion at a rate up to 20 mL/h/site, which, if well tolerated, can be increased to 35 mL/h/site, with further increases at the discretion of the patient and physician [8].

Previous studies of IgPro20 and other SCIG preparations, as well as real-life clinical experience, have suggested the possibility of using higher IgPro20 infusion parameters than the currently approved levels in the USA [3, 16–20]. For instance, an infusion volume of 40 mL/site and a flow rate of 50 mL/h/site were allowed in the US and EU phase III extension studies of IgPro20 [3]. Another prospective study reported the use of infusion parameters of up to 60 mL/site and 60 mL/h/site for the administration of a different 20% SCIG product [16]. However, the safety and tolerability of higher-than-approved parameters in the USA have not been evaluated in a systematic manner in a prospective clinical trial (see Fig. S1 for a systematic literature search of clinical trials evaluating pump-assisted and manual push infusion of SCIG). The aim of the present study was to determine the safety and tolerability of IgPro20 administration using increasing infusion parameters via pump-assisted and manual push administration in patients with PID. Here, we describe the overall study design and report the results obtained from the pump-assisted cohorts. The results of the manual push cohort are reported in the accompanying manuscript [21].

## Methods

### Study Design and Patients

The Hizentra^®^ Label Optimization (HILO) study was a multicenter, open-label, parallel-arm, non-randomized phase IV trial using a forced upward titration design to evaluate the safety and tolerability of IgPro20 at high infusion parameters in patients with PID (NCT03033745).

The study was conducted at 12 sites in the USA and Canada. Male and female patients with PID (e.g., with diagnosis of common variable immunodeficiency [CVID] or X-linked agammaglobulinemia [XLA] as defined by the Pan-American Group for Immunodeficiency and the European Society for Immunodeficiencies or by the International Union of Immunological Societies Expert Committee) were included in the study. Patients were enrolled and assigned to one of three cohorts: Pump-Assisted Volume Cohort, Pump-Assisted Flow Rate Cohort, or Manual Push Flow Rate Cohort (see [21] in this issue for details of the Manual Push Flow Rate Cohort). To be enrolled, patients had to meet cohort-specific inclusion criteria. Patients who were receiving a stable dose of IgPro20 therapy at the following infusion parameters for ≥ 1 month prior to study day 1 were included:

- Pump-Assisted Volume Cohort: IgPro20 at a volume of 25 mL per injection site and a total weekly volume of ≥ 50 mL
- Pump-Assisted Flow Rate Cohort: IgPro20 at a flow rate of 25 mL/h per injection site
- Manual Push Flow Rate Cohort: IgPro20 at a flow rate of ~ 0.5 mL/min (equivalent to 25–30 mL/h) per injection site.

Patients with known or suspected hypersensitivity to IgPro20, with ongoing serious bacterial infections at screening, or with other significant medical conditions were excluded from the study.

**Fig. 1:**
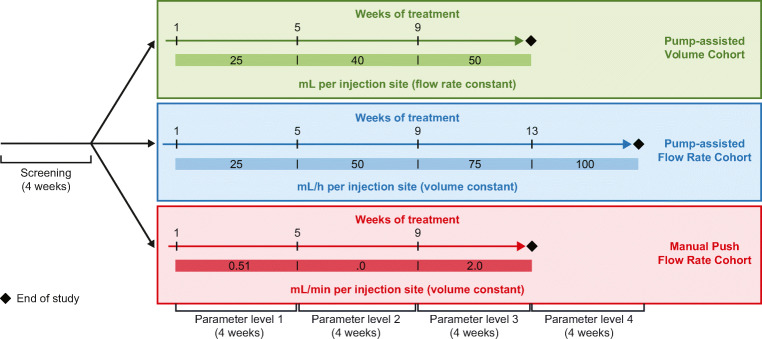
HILO study design

The study contained a screening period of up to 28 days (4 weeks) followed by an active treatment period of 12 weeks in the Pump-Assisted Volume Cohort and 16 weeks in the Pump-Assisted Flow Rate Cohort (Fig. 1). Each cohort evaluated escalating IgPro20 infusion parameter levels (volumes or flow rates) for 4 weeks before switching to the next parameter level. In the Pump-Assisted Volume Cohort, the volume of IgPro20 infusions per injection site was 25–50 mL; in the Pump-Assisted Flow Rate Cohort, the flow rate per injection site was 25–100 mL/h (Fig. 1). Only 1 injection site per patient was used to evaluate the infusion parameters. IgPro20 infusions were administered once per week. The IgPro20 dose for each patient remained unchanged during the study. Treatments were administered either at the study site (the first infusion for each 4-week period) or at home (the remaining infusions for each 4-week period).

### Safety Assessments

#### Definitions

A patient was considered a responder for each infusion parameter level in the pump-assisted cohorts upon completion of ≥ 3 valid weekly infusions for that infusion parameter level (i.e., completion of ≥ 75% of planned infusions). An infusion was considered valid if the patient had completed ≥ 95% of the planned dose at the scheduled volume or flow rate without interruption or decrease during infusion for any reason, including mechanical problems.

An infusion parameter level was considered successful if the response rate was ≥ 33% for that level:

$$ \mathrm{Response}\ \mathrm{rate}\ \left(\%\right)=\frac{100\ast \left(\mathrm{Number}\ \mathrm{of}\ \mathrm{responders}\ \mathrm{for}\ \mathrm{in}\mathrm{fusion}\ \mathrm{parameter}\ \mathrm{in}\ \mathrm{cohort}\right)\ }{\mathrm{Number}\ \mathrm{of}\ \mathrm{patients}\ \mathrm{in}\ \mathrm{the}\ \mathrm{safety}\ \mathrm{analysis}\ \mathrm{set}\ \mathrm{for}\ \mathrm{cohort}\ } $$

In the absence of regulatory guidance, the threshold of ≥ 33% was based on our analysis of previous IgPro20 clinical studies and consultations with physicians in the field of PID.

#### Responder Analysis

The primary study endpoint was to determine responder rates at every infusion parameter level. Each infusion parameter level was tested for 4 weeks, and then responders were switched to the next level. Non-responders continued IgPro20 administration at the highest previously tolerated infusion parameter level for the remainder of the study period, and safety data were collected.

#### Safety and Tolerability

Duration of exposure was calculated irrespective of response status (i.e., duration of exposure within an infusion parameter level was considered for both responders and non-responders).

Secondary endpoints included the safety and tolerability of IgPro20 infusions with high infusion parameters. Treatment-emergent AEs (TEAEs) were evaluated in each cohort. Patients reported AEs in eDiaries/backup paper diaries, by phone call, or during site visits. All reported events were evaluated by medical site staff to determine if the event constituted an AE; if so, the AE was entered into the electronic case report form. The number of patients who discontinued study drug administration due to TEAEs was also evaluated. TEAEs were summarized using data up to a patient’s non-response at a particular parameter level. Safety data collected after non-response were excluded from analyses of TEAEs carried out under forced upward titration conditions. The frequency and intensity of TEAEs were characterized.

Tolerability was defined as the number of infusions achieved without severe local reactions divided by the total number of infusions, irrespective of infusion validity; tolerability of 100% corresponded to the most favorable tolerability outcome with no severe local reactions.

### Efficacy Assessments

The exploratory objective of the study included assessment of the serum IgG concentrations as a surrogate efficacy endpoint at baseline (day 1) and at the end-of-study visit.

### Statistical Analysis

All efficacy and safety analyses were performed in the safety analysis set, which comprised all patients who received ≥ 1 dose or a partial dose of IgPro20 in the study. Continuous variables were summarized in terms of the number of observations, mean, standard deviation (SD), median, minimum, and maximum. Categorical variables were summarized using frequency counts and percentages. Percentages were based on non-missing values. Exploratory endpoints were presented as mean and SD. Responder rates were summarized by overall patients at a parameter level, by age (≤ 17 years, > 17 years), and by body mass index (BMI; < 30 kg/m^2^, ≥ 30 kg/m^2^). Statistical analyses were performed using SAS version 9.3 (SAS Institute Inc., Cary, NC, USA).

Infusion compliance rates were analyzed and summarized by infusion parameter level and by cohort. Infusion compliance refers to adherence with the planned dose and either the volume or flow rate scheduled during the study. Overall infusion compliance was determined based on patient diaries of administered infusions and calculated as a percentage:

$$ \mathrm{Overall}\ \mathrm{compliance}\ \left(\%\right)=\frac{100\ast \left(\mathrm{Cumulative}\ \mathrm{actual}\ \mathrm{dose}\ \mathrm{over}\ \mathrm{all}\ \mathrm{infusions}\right)}{\mathrm{Cumulative}\ \mathrm{planned}\ \mathrm{dose}\ \mathrm{over}\ \mathrm{all}\ \mathrm{infusions}} $$

## Results

### Patient Disposition and Demographics

**Fig. 2:**
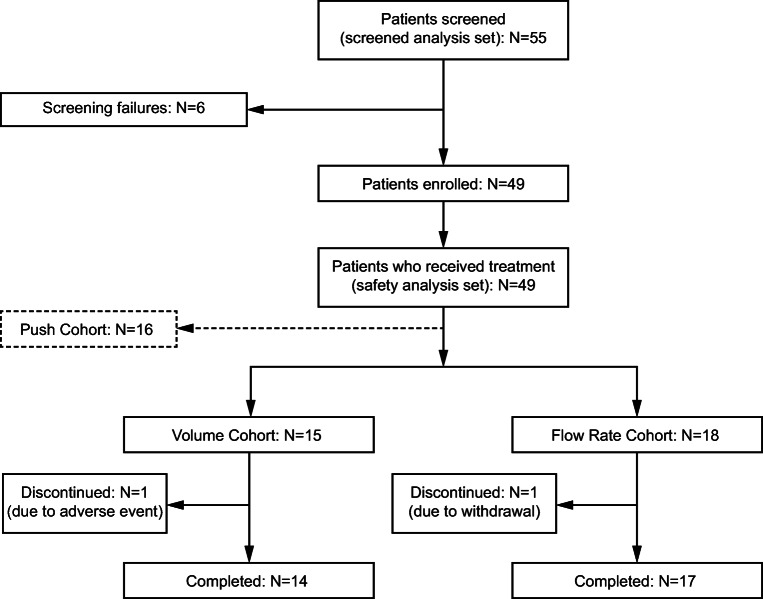
Patient disposition. Disposition of patients in the HILO study with a focus on the pump-assisted cohorts

A total of 15 patients were included in the Volume Cohort, 14 of whom completed the study (Fig. 2). One patient discontinued due to an AE at the 25-mL volume level. The Flow Rate Cohort included 18 patients, 17 of whom completed the study. One patient withdrew at the 25-mL/h flow rate level.

The proportion of males and females in the two pump-assisted cohorts was similar (Table 1). Mean (SD) age of these patients was 49.1 (14.2) and 26.7 (24.5) years in the Volume Cohort and Flow Rate Cohort, respectively. Ten patients (55.6%) in the Flow Rate Cohort were ≤ 17 years old (Table 1). Due to the eligibility requirement for patients in the Volume Cohort to have a total weekly IgPro20 dose of ≥ 50 mL, there were no patients aged ≤ 17 years in this cohort, as younger patients would have had a lower body weight, resulting in lower volumes at the same dose in mg/kg. The median BMI was 27.7 kg/m^2^ in the Volume Cohort and 22.3 kg/m^2^ in the Flow Rate Cohort; 7 patients (21.2%), 4 in the Volume Cohort and 3 in the Flow Rate Cohort, were considered obese (BMI ≥ 30 kg/m^2^). Overall, these differences in age and BMI between the cohorts were not considered clinically relevant nor were they anticipated to impact treatment outcomes.

**Table 1:** Patient demographics and baseline characteristics (safety analysis set)

Parameter	Volume Cohort (*N* = 15)	Flow Rate Cohort (*N* = 18)
Age, years		
Mean (SD)	49.1 (14.2)	26.7 (24.5)
Median (min, max)	50.0 (19, 75)	15.0 (2, 75)
Age category, years		
≤ 17	0^a^	10 (55.6)
< 16	0^a^	9 (50.0)
> 17	15 (100.0)	8 (44.4)
Sex, *n* (%)		
Male	6 (40.0)	8 (44.4)
Female	9 (60.0)	10 (55.6)
Race, *n* (%)		
White	14 (93.3)	16 (88.9)
American Indian or Alaska Native	0	1 (5.6)
Black or African American	0	1 (5.6)
Multiple	1 (6.7)	0
Weight, kg		
Mean (SD)	80.1 (21.0)	52.6 (26.1)
Median (min, max)	71.4 (55.8, 143.1)	59.0 (11.3, 88.8)
BMI, kg/m^2^		
Median (min, max)	27.7 (23.2, 58.1)	22.3 (13.4, 31.4)
BMI category, *n* (%)		
< 30 kg/m^2^	11 (73.3)	15 (83.3)
≥ 30 kg/m^2^	4 (26.7)	3 (16.7)
Concomitant diseases (≥ 4 patients in each cohort), *n* (%)		
Any concomitant disease	15 (100)	18 (100)
Asthma	9 (60.0)	7 (38.9)
Rhinitis allergic	7 (46.7)	7 (38.9)
Immunodeficiency disease, *n* (%)		
Common variable immunodeficiency	11 (73.3)	8 (44.4)
Congenital agammaglobulinemia	1 (6.7)	1 (5.6)
Other immunodeficiency^b^	3 (20.0)	9 (50.0)
Time since first PID diagnosis, years		
Mean (SD)	11.1 (13.0)	5.2 (6.0)
Median (min, max)	5.0 (0.8, 45.0)	2.3 (0.2, 23.0)
IgG levels at time of first PID diagnosis, g/L		
*n*	11	14
Mean (SD)	4.3 (2.4)	4.1 (2.8)
Median (min, max)	5.5 (1.0, 7.0)	4.7 (0.1, 9.2)
Pre-study IgG trough levels, g/L		
*n*	15	18
Mean (SD)	11.2 (2.8)	9.6 (3.0)
Median (min, max)	11.6 (6.9, 16.1)	9.9 (1.5, 14.2)

^a^Patients were required to have a total weekly IgPro20 dose of ≥ 50 mL to be eligible for enrollment in this cohort. This dose is not pertinent for pediatric patients because IgPro20 doses need to be adjusted based on patient body weight

^b^Other immunodeficiency category includes combined immunodeficiency, specific antibody deficiency, hypogammaglobulinemia, IgG deficiency, Bruton’s agammaglobulinemia, polysaccharide non-response immunodeficiency, and ZAP70 immunodeficiency

*BMI* body mass index, *IgG* immunoglobulin G, *PID* primary immunodeficiency, *SD* standard deviation

### Responder Analysis

**Fig. 3:**
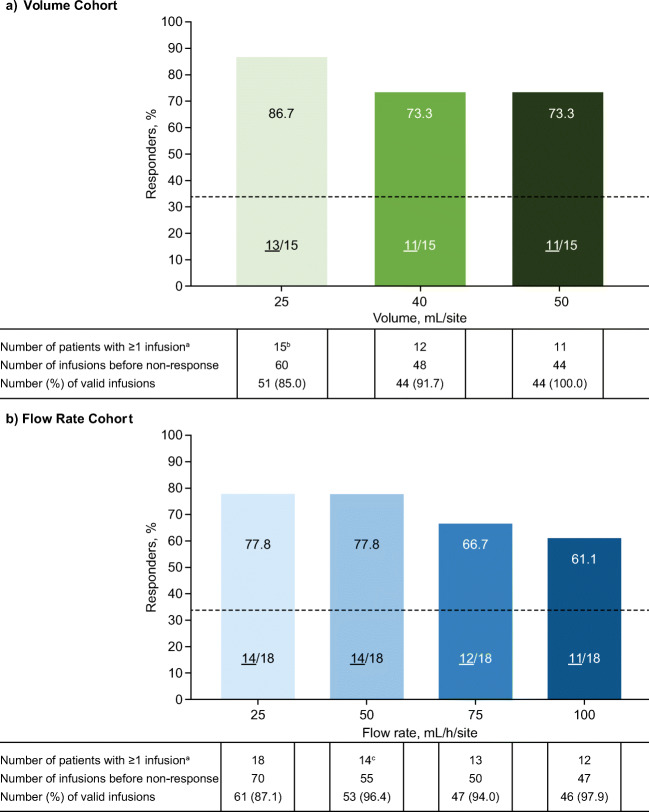
Responder analysis (safety analysis set). Dashed line indicates ≥ 33% prespecified success criterion. The number of responders for each parameter level is shown at the bottom of each bar (underlined). ^a^Before start date of non-response. ^b^One patient discontinued after completing the 25-mL volume level due to a related TEAE of injection site pain. This patient fulfilled the criteria for 3 valid infusions, and data up to the discontinuation were included in the analysis. ^c^One patient had 3 valid infusions at the 50-mL/h flow rate level but returned to the 25-mL/h level due to intolerance without trying the 75-mL/h level. This patient was classified as responder for the 50-mL/h flow rate level. TEAE, treatment-emergent adverse event

In the Volume Cohort, responder rates were 86.7% at the 25-mL volume level and 73.3% at both the 40- and 50-mL/injection site levels, meeting the prespecified success criterion of ≥ 33% for all infusion parameter levels. The percentage of valid infusions before non-response ranged between 85.0 and 100.0% (Fig. 3a). Responder rates at each volume level were similar between non-obese (*n* = 11; 10 responders [90.9%] at 25 mL; 8 responders [72.7%] at 40 mL and 50 mL) and obese (*n* = 4; 3 responders [75.0%] at all levels) patients in the Volume Cohort.

In the Flow Rate Cohort, responder rates were 77.8% at 25 and 50 mL/h, 66.7% at 75 mL/h, and 61.1% at 100 mL/h, meeting the prespecified success criterion of ≥ 33% for all infusion parameter levels. The percentage of valid infusions before non-response ranged between 87.1 and 97.9% (Fig. 3b). There were no substantial differences in responder rates at each flow rate level between patients aged ≤ 17 years (*n* = 10; 80.0% response rate at 25 and 50 mL/h; 70.0% at 75 mL/h; 60% at 100 mL/h) and those aged > 17 years (*n* = 8; 75.0% at 25 and 50 mL/h; 62.5% at 75 and 100 mL/h). Responder rates in non-obese patients (*n* = 15) were 86.7% at 25 and 50 mL/h, 73.3% at 75 mL/h, and 66.7% at 100 mL/h; the responder rate was 33.3% (1 responder) at all flow rate levels in obese patients (*n* = 3).

### Effect of High Infusion Parameters on Number of Injection Sites and Infusion Time

**Fig. 4:**
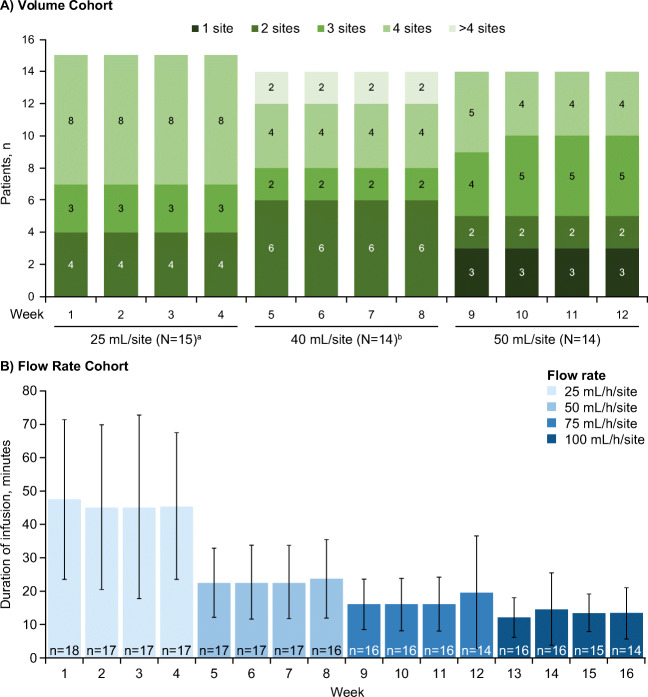
Number of injection sites and infusion duration with increasing infusion parameters (safety analysis set). Only 1 injection site per patient was used to evaluate the infusion parameters. Only infusions that were administered at the planned parameter level for the particular week are included, irrespective of the patient’s response status. The duration of infusion per patient per week is calculated as the sum of all individual durations of single pump-assisted infusions given in the respective week, even if the infusions are overlapping. (**a**) Reduction in the number of injection sites in the Pump-Assisted Volume Cohort (number of patients who used 1, 2, 3, 4, or > 4 sites per week); (**b**) Reduction in the duration of infusion in the Pump-Assisted Flow Rate Cohort (mean ± SD). ^a^One patient discontinued after completing the 25-mL level due to a related TEAE of injection site pain. ^b^Two patients were non-responders at the 40-mL level and used 7 injection sites for each of their weekly infusions at that level. SD, standard deviation; TEAE, treatment-emergent adverse event

In the Volume Cohort, the median weekly number of injection sites decreased from 4 sites at the 25-mL level to 3 sites at the 40-mL and 50-mL levels. Furthermore, the number of patients who used ≥ 4 injection sites per week decreased by 50% (from 8 to 4 patients) from week 1, 25 mL, to week 12, 50 mL (Fig. 4a). Of the 14 patients who completed the study, 9 (64.3%) used fewer injection sites per week at week 12 compared with week 1: 4 patients reduced from 4 to 3 injection sites, 2 patients reduced from 3 to 2 sites, and 3 patients reduced from 2 sites to 1 site. In the Flow Rate Cohort, the mean (SD) weekly infusion time decreased almost fourfold from 47.3 (23.93) min at week 1, 25 mL/h, to 13.1 (7.69) min at week 16, 100 mL/h (Fig. 4b).

### Infusion Compliance

In the Volume Cohort, overall infusion compliance was ≥ 90% (Table 2). In the Flow Rate Cohort, 83.3% of patients had infusion compliance ≥ 90%, with < 90% overall compliance reported in 3 patients (Table 2). Of these patients, 1 patient discontinued following 2 infusions at the 25-mL/h level, 1 patient received 3 out of 4 planned infusions at the 50-mL/h level and switched back to 25 mL/h due to poor tolerability, and 1 patient received 2 infusions at the 100-mL/h level and switched back to 75 mL/h due to severe injection site pain. Five patients had a compliance of < 90% at some point during the study (Table 2).

**Table 2:** Infusion compliance (safety analysis set)

Pump-Assisted Volume Cohort	25 mL (*N* = 15)	40 mL (*N* = 2)	50 mL (*N* = 11)	
Overall compliance (administered dose/planned dose, %)				
Mean (SD)	100.3 (1.2)	100.4 (1.3)	100.4 (1.4)	
Median (min, max)	100.0 (99.8, 104.5)	100.1 (99.8, 104.5)	100.0 (99.8, 104.5)	
Compliance level, *n* (%)				
< 90%	0	0	0	
≥ 90%	15 (100.0)	12 (100.0)	11 (100.0)	
Pump-Assisted Flow Rate Cohort	25 mL/h (*N* = 18)	50 mL/h (*N* = 14)	75 mL/h (*N* = 13)	100 mL/h (*N* = 12)
Overall compliance (administered dose/planned dose, %)				
Mean (SD)	97.3 (10.7)	98.1 (6.8)	96.0 (8.8)	98.5 (16.8)
Median (min, max)	100.0 (54.5, 100.3)	100.0 (74.7, 100.3)	100.0 (75.0, 100.4)	100.0 (50.1, 124.7)
Compliance level, *n* (%)				
< 90%	1 (5.6)	1 (7.1)	2 (15.4)	1 (8.3)
≥ 90%	17 (94.4)	13 (92.9)	11 (84.6)	11 (91.7)

*SD* standard deviation

### Safety and Tolerability

In the Volume Cohort, individual weekly IgPro20 doses ranged from 104.8 to 324.7 mg/kg; median weekly doses for all patients in the cohort ranged from 162.0 to 170.6 mg/kg. In the Flow Rate Cohort, individual weekly IgPro20 doses ranged from 89.5 to 190.5 mg/kg; median weekly doses for all patients in the cohort ranged from 96.7 to 177.0 mg/kg, with median weekly volumes from 10 to 65 mL. Two pediatric patients in this cohort (≤ 17 years category) received very low volumes of 10 mL per week due to low body weight.

Overall, including TEAEs occurring after non-response, 8 patients (53.3%) in the Volume Cohort experienced 25 TEAEs across all infusion flow rates, with a rate of 0.145 TEAEs per infusion. In the Flow Rate Cohort, 12 patients (66.7%) reported 62 TEAEs (0.228 TEAEs per infusion).

Excluding TEAEs occurring after non-response, in the Volume Cohort, 21 TEAEs were reported in 7 patients (46.7%). Of these, 12 TEAEs in 4 patients (26.7%) were deemed related to study drug administration. The overall TEAE rate per infusion was 0.138 (0.079 for related TEAEs) (Table 3). The rate of any TEAE was lower at the 50-mL level compared with the 25- and 40-mL levels, mostly due to the fact that there were no mild TEAEs at the 50-mL level. The rate of moderate TEAEs remained below 0.1 at all infusion levels. No severe TEAEs were reported in this cohort. One treatment-related TEAE (injection site pain) led to discontinuation by 1 patient after completing the 25-mL level (Table 3).

**Table 3:** Treatment-emergent adverse events under forced upward titration conditions in Pump-Assisted Volume Cohort (safety analysis set)^a^

Pump-Assisted Volume Cohort	25 mL (*N* = 15; Inf = 60)		40 mL (*N* = 12; Inf = 48)		50 mL (*N* = 11; Inf = 44)	
	*n* (%)	*E* (Rate)	*n* (%)	*E* (Rate)	*n* (%)	*E* (Rate)
Any TEAE	4 (26.7)	11 (0.183)	4 (33.3)	9 (0.188)	1 (9.1)	1 (0.023)
Treatment related	3 (20.0)	9 (0.150)	1 (8.3)	3 (0.063)	0	0
Intensity of TEAEs						
Mild	3 (20.0)	10 (0.167)	2 (16.7)	6 (0.125)	0	0
Moderate	1 (6.7)	1 (0.017)	2 (16.7)	3 (0.063)	1 (9.1)	1 (0.023)
Severe	0	0	0	0	0	0
Serious TEAEs	0	0	0	0	0	0
Deaths	0	0	0	0	0	0
Study discontinuation due to TEAE	1 (6.7)	1 (0.017)	0	0	0	0
Treatment related	1 (6.7)	1 (0.017)	0	0	0	0
Study drug withdrawal due to TEAE	1 (6.7)	2 (0.033)	0	0	0	0
Treatment related	1 (6.7)	2 (0.033)	0	0	0	0
Local TEAEs	3 (20.0)	9 (0.150)	1 (8.3)	3 (0.063)	0	0
Treatment related	3 (20.0)	9 (0.150)	1 (8.3)	3 (0.063)	0	0
Most common (> 1 event at any infusion volume) TEAEs by preferred term						
Injection site swelling	1 (6.7)	4 (0.067)	0	0	0	0
Injection site erythema	1 (6.7)	1 (0.017)	1 (8.3)	2 (0.042)	0	0
Injection site pain	1 (6.7)	2 (0.033)	0	0	0	0
Most common (> 1 event at any infusion volume) treatment-related TEAEs by preferred term						
Injection site swelling	1 (6.7)	4 (0.067)	0	0	0	0
Injection site erythema	1 (6.7)	1 (0.017)	1 (8.3)	2 (0.042)	0	0
Injection site pain	1 (6.7)	2 (0.033)	0	0	0	0

Rate = number of events/total number of infusions prior to patient’s start date of non-response

^a^Excludes TEAEs occurring after non-response

*E* number of events, *Inf* infusions, *n* number of patients, *TEAE* treatment-emergent adverse event

Excluding TEAEs occurring after non-response, in the Flow Rate Cohort, 48 TEAEs were reported in 12 patients (66.7%). Of these, 35 TEAEs in 8 patients (44.4%) were considered related to the study medication (Table 4). A decrease in the frequency of both mild and moderate TEAEs was observed with increasing infusion flow rate. The overall TEAE rate per infusion was 0.216 (0.158 for related TEAEs) (Table 4).

**Table 4:** Treatment-emergent adverse events under forced upward titration conditions in Pump-Assisted Flow Rate Cohort (safety analysis set)^a^

Pump-Assisted Flow Rate Cohort	25 mL/h (*N* = 18; Inf = 70)		50 mL/h (*N* = 14; Inf = 55)		75 mL/h (*N* = 13; Inf = 50)		100 mL/h (*N* = 12; Inf = 47)	
	*n* (%)	*E* (Rate)	*n* (%)	*E* (Rate)	*n* (%)	*E* (Rate)	*n* (%)	*E* (Rate)
Any TEAE	7 (38.9)	23 (0.329)	4 (28.6)	14 (0.255)	3 (23.1)	7 (0.140)	3 (25.0)	4 (0.085)
Treatment related	5 (27.8)	21 (0.300)	3 (21.4)	9 (0.164)	1 (7.7)	2 (0.040)	2 (16.7)	3 (0.064)
Intensity of TEAEs								
Mild	5 (27.8)	19 (0.271)	4 (28.6)	14 (0.255)	1 (7.7)	4 (0.080)	1 (8.3)	1 (0.021)
Moderate	3 (16.7)	4 (0.057)	0	0	2 (15.4)	3 (0.060)	1 (8.3)	1 (0.021)
Severe	0	0	0	0	0	0	1 (8.3)	2 (0.043)
Serious TEAEs	0	0	0	0	0	0	0	0
Deaths	0	0	0	0	0	0	0	0
Study discontinuation due to TEAE	0	0	0	0	0	0	0	0
Study drug withdrawal due to TEAE	0	0	0	0	0	0	0	0
Local TEAEs	5 (27.8)	20 (0.286)	3 (21.4)	8 (0.145)	1 (7.7)	2 (0.040)	1 (8.3)	1 (0.021)
Treatment related	5 (27.8)	20 (0.286)	3 (21.4)	8 (0.145)	0	0	1 (8.3)	1 (0.021)
Most common (> 1 event at any flow rate) TEAEs by preferred term								
Injection site pain	2 (11.1)	7 (0.100)	2 (14.3)	5 (0.091)	0	0	1 (8.3)	1 (0.021)
Injection site erythema	3 (16.7)	8 (0.114)	0	0	0	0	0	0
Injection site pruritus	2 (11.1)	2 (0.029)	1 (7.1)	2 (0.036)	0	0	0	0
Injection site swelling	2 (11.1)	3 (0.043)	0	0	0	0	0	0
Injection site hemorrhage	10	0	0	0	1 (7.7)	2 (0.040)	0	0
Headache		0	2 (14.3)	2 (0.036)	1 (7.7)	1 (0.020)	1 (8.3)	1 (0.021)
Most common (> 1 event at any flow rate) treatment-related TEAEs by preferred term								
Injection site pain	2 (11.1)	7 (0.100)	2 (14.3)	5 (0.091)	0	0	1 (8.3)	1 (0.021)
Injection site erythema	3 (16.7)	8 (0.114)	0	0	0	0	0	0
Injection site pruritus	2 (11.1)	2 (0.029)	1 (7.1)	2 (0.036)	0	0	0	0
Injection site swelling	2 (11.1)	3 (0.043)	0	0	0	0	0	0

Rate = number of events/total number of infusions prior to patient’s start date of non-response

^a^Excludes TEAEs occurring after non-response

*E* number of events, *Inf* infusions, *n* number of patients, *TEAE* treatment-emergent adverse event

The most frequent TEAEs across both cohorts were injection site pain, injection site erythema, and injection site swelling (Table 3 and Table 4). No deaths or serious TEAEs were reported in either cohort (Table 3 and Table 4).

There were no clinically meaningful differences in the frequency, type, or intensity of TEAEs during the study in either cohort. Within the parameters tested in this study, the rate and intensity of TEAEs did not increase with increasing infusion volume or flow rate per injection site.

In the Volume Cohort, tolerability was 100% for all volume levels. In the Flow Rate Cohort, tolerability was 100% for the 25-, 50-, and 75-mL/h flow rates and 98% for the 100-mL/h flow rate. One patient (6 years of age) experienced a severe TEAE (injection site pain) within 72 h after the infusion at the 100-mL/h level, which resolved within a day.

### Serum IgG Trough Concentrations

In both cohorts, serum IgG trough levels were similar between day 1 and the end of the study. Mean (SD) IgG levels were 10.19 (2.35) g/L on day 1 and 10.96 (2.42) g/L at the end of the study in the Volume Cohort and 10.40 (2.10) g/L on day 1 and 10.62 (1.87) g/L at the end of the study in the Flow Rate Cohort.

## Discussion

Our study evaluated the feasibility of higher than currently approved infusion parameters of IgPro20 using a forced upward titration design. Responder rates of 73.3% at a 50-mL infusion volume and 61.1% at a 100-mL/h infusion flow rate were observed in patients with PID who had prior experience with pump-assisted infusions. These responder rates were approximately twofold higher than the prespecified success criterion of ≥ 33%. There were no clinically meaningful differences in responder rates between age subgroups in the Flow Rate Cohort nor were there clinically meaningful differences between obese and non-obese patients in the Volume Cohort. For patients with PID, age does not influence the clinical efficacy or safety of IgPro20, as therapy has been shown to be equally effective and have a similar safety profile in pediatric, adolescent, and adult patients. In the Flow Rate Cohort, the responder rate was lower in obese patients. However, the latter results must be interpreted with caution due to the small numbers of obese patients in both cohorts. Other studies have found comparable efficacy and safety of IgPro20 in obese and non-obese patients [10, 22]. Thus, any overall differences in age and BMI among pump-assisted cohorts would not be expected to affect treatment outcomes. Further evaluation is needed to specifically assess the tolerability of high infusion volumes and flow rates in underweight (BMI ≤ 18 kg/m^2^) patients.

Importantly, increasing infusion parameters did not negatively impact the safety and tolerability of IgPro20. The TEAE rate per infusion for all TEAEs, as well as for local reactions, was low across infusion parameter levels. The TEAE rates per infusion observed in our study were also considerably lower than the rates observed in most previous IgPro20 studies in patients with PID. The TEAE rate per infusion was 0.288 in the European pivotal phase III study, 0.773 in the US pivotal phase III study, and 0.661 in the US phase III extension study [2, 3, 23]. This result might be explained by the prior experience of enrolled patients with the infusion technique and the fact that all patients were receiving IgPro20 prior to this study. A numerical decrease in TEAE rate with increasing volume in the Volume Cohort and with increasing flow rate in the Flow Rate Cohort was observed. This trend might be due to negative selection of patients with a lower tolerability threshold after their non-response at a certain parameter level; patients who completed the highest volume or flow rate levels may have had higher tolerability levels, objectively or subjectively, compared with those who were non-responders at the highest parameters. However, any apparent tendencies in TEAE rates observed in this study should be interpreted with caution, as the low overall number of TEAEs does not allow for reliable evaluation.

Excellent dose compliance was observed in both the Volume and Flow Rate Cohorts, indicating that high infusion parameter levels did not negatively affect the therapy adherence rates in the study population. No clinically meaningful differences were observed in serum IgG trough concentrations between the start and end of the study in either cohort, suggesting that, for the same total weekly dose, increased volume or flow rate of IgPro20 infusions did not affect serum IgG trough concentrations.

High volumes and high flow rates of IgPro20 infusions reduced the number of injection sites and the duration of infusions, respectively. However, results regarding the number of injection sites should be interpreted conservatively: firstly, per protocol, only 1 injection site had to be evaluated for higher volume; secondly, individual weekly doses may have prevented some patients from reducing the number of injection sites because of the number of vials required to administer the dose. Despite these limitations, the majority of patients used fewer injection sites per week at the end of the study compared with week 1. Therefore, high volumes and high flow rates would be expected to improve overall administration convenience and provide patients more choices to make individualized treatment decisions.

The patient population in the HILO study is representative of the general population of patients with PID in terms of specific immunodeficiencies, the range of patients’ age and BMI, and IgG levels at study entry. This study included a majority of patients with CVID, as well as patients with congenital agammaglobulinemia disorders, such as XLA. The distribution of PID diseases herein is consistent with previous studies of IgPro20 in both US and European patient populations [2, 23]. The age range of patients in the HILO study was 2–75 years, similar to the ranges in previous IgPro20 studies (3–60 and 5–72 years [2, 23]). The overall proportion of pediatric (≤ 15 years) patients in the HILO study was similar to a previous study of IgPro20 in the USA [23]; however, all patients aged ≤ 15 years in the current study happened to be enrolled in the Pump-Assisted Flow Rate Cohort. In general, the median (min, max) BMI of patients in the HILO study was somewhat higher than that of patients in a previous European IgPro20 study (26.1 [13.4–58.1] vs 20.1 [12–26] kg/m^2^ [2]). Finally, the mean (SD) IgG level of all patients at HILO study entry was 9.9 (2.7) g/L compared with 10.1 (2.6) g/L in a previous US IgPro20 study [23] and 7.5 (1.6) g/L in a European IgPro20 study [2]. Therefore, despite the small number of patients in each cohort of the HILO study, the overall HILO patient population is representative of the general population of patients with PID.

The present study aimed to address the gap in the existing landscape of standardized study designs and/or study designs recommended by regulatory guidance documents that evaluate the safety and tolerability of IgG infusions in IgG replacement therapy. Registrational IgG clinical studies do not routinely investigate infusion parameters that are higher than those used conventionally, which may reduce the time needed for infusion and the number of injection sites, improving the health-related quality of life of patients with a life-long therapy [24–26].

In conclusion, our prospective study, conducted under forced upward titration conditions, demonstrated that pump-assisted IgPro20 infusion volumes of up to 50 mL and flow rates of up to 100 mL/h per injection site are well-tolerated in the majority of treatment-experienced patients with PID. These findings are consistent with previous studies of high infusion parameters, either with IgPro20 or other 20% SCIG preparations administered via pump-assisted infusions [3, 16, 18–20]. This study is the first prospective study that applied a forced upward titration study design to evaluate individual safety and tolerability levels of pump-assisted and manual push SCIG infusion parameters. These results may help clinicians and patients make informed decisions about individualizing SCIG therapy.

## Supplementary Information

ESM 1 (DOCX 95.9 kb).
