# Supplementary material for: Safety and Tolerability of Subcutaneous IgPro20 at High Infusion Parameters in Patients with Primary Immunodeficiency: Findings from the Pump-Assisted Administration Cohorts of the HILO Study
Source: J Clin Immunol. 2021 Jan 6;41(2):458–69. doi: 10.1007/s10875-020-00912-5 (PMC7858210; doi:10.1007/s10875-020-00912-5)
Supplement: Supplementary file 1 — (DOCX 95.9 kb). [file 10875_2020_912_MOESM1_ESM.docx]

**Fig. S1 Flowchart for systematic literature search of clinical trials evaluating pump-assisted and manual push SCIG infusions**


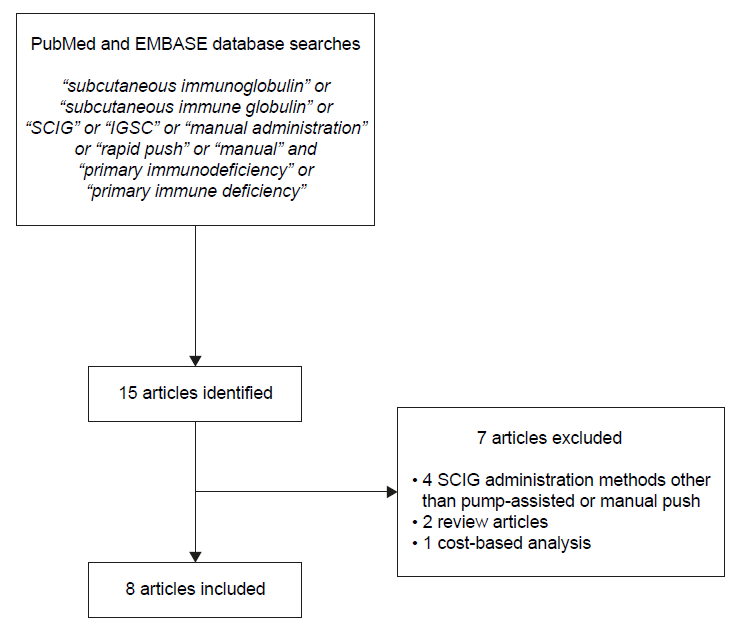


In order to fully characterize the current landscape regarding clinical studies conducted with pump-assisted and manual push infusion, a systematic search of the currently published literature was performed. The PubMed and EMBASE databases were searched from January 2009 until April 2019 for full text publications reporting actual treated patients with PID, using the following search terms: “subcutaneous immunoglobulin” or “subcutaneous immune globulin” or “SCIG” or “IGSC” or “manual administration” or “rapid push” or “manual” and “primary immunodeficiency” or “primary immune deficiency.” In total, 15 studies were identified [1-15], of which 8 studies met the inclusion criteria [1-8]. Among the studies included in the analysis, a range of SCIG therapies were administered to patients with PID using manual push or pump-assisted techniques [1, 3, 5-8, 2, 4]. The duration of the treatment varied across the studies, ranging from 1.5 to 63.0 months [1-8]. Mean IgG levels reported were similar between push and pump techniques and were also similar across the studies [1, 5-8, 2, 4]. The comparative rates of infusion site reactions reported in the literature for both manual push and pump-assisted SCIG administration vary [5, 7, 2, 1, 8]. This is most likely due to differences in study design, scales, timepoints, and report methodology. Across the identified studies, the average flow rates using a manual push technique ranged from 10 to 60 mL/h per injection site [1, 4]. The studies reported no major safety concerns, as most AEs were local reactions of mild severity [1-8]. Generally, for pump-assisted techniques, volumes of up to 50 mL per injection site [1, 16] and infusion flow rates of 20 to 50 mL/h per injection site are used [5, 17, 18, 16].

AE, adverse event; IGSC, subcutaneous immunoglobulin G; SCIG, subcutaneous immunoglobulin G; PID, primary immunodeficiency.

# References

1. Bienvenu B, Cozon G, Mataix Y, Lachaud D, Alix A, Hoarau C et al. Rapid push vs pump-infused subcutaneous immunoglobulin treatment: a randomized crossover study of quality of life in primary immunodeficiency patients. J Clin Immunol. 2018;38(4):503-12. doi:10.1007/s10875-018-0507-x.

2. Misbah S, Sturzenegger MH, Borte M, Shapiro RS, Wasserman RL, Berger M et al. Subcutaneous immunoglobulin: opportunities and outlook. Clin Exp Immunol. 2009;158 Suppl 1:51-9. doi:10.1111/j.1365-2249.2009.04027.x.

3. Paris K, Haddad E, Borte M, Brodszki N, Derfalvi B, Marodi L et al. Tolerability of subcutaneous immunoglobulin 20%, Ig20Gly, in pediatric patients with primary immunodeficiencies. Immunotherapy. 2019;11(5):397-406. doi:10.2217/imt-2018-0088.

4. Patel NC, Gallagher JL, Ochs HD, Atkinson TP, Wahlstrom J, Dorsey M et al. Subcutaneous immunoglobulin replacement therapy with Hizentra^®^ is safe and effective in children less than 5 years of age. J Clin Immunol. 2015;35(6):558-65. doi:10.1007/s10875-015-0190-0.

5. Shapiro R. Subcutaneous immunoglobulin therapy by rapid push is preferred to infusion by pump: a retrospective analysis. J Clin Immunol. 2010;30(2):301-7. doi:10.1007/s10875-009-9352-2.

6. Shapiro R. Subcutaneous immunoglobulin (16 or 20%) therapy in obese patients with primary immunodeficiency: a retrospective analysis of administration by infusion pump or subcutaneous rapid push. Clin Exp Immunol. 2013;173(2):365-71. doi:10.1111/cei.12099.

7. Shapiro RS. Subcutaneous immunoglobulin therapy given by subcutaneous rapid push vs infusion pump: a retrospective analysis. Ann Allergy Asthma Immunol. 2013;111(1):51-5. doi:10.1016/j.anai.2013.04.015.

8. Shapiro RS. Subcutaneous immunoglobulin: rapid push vs. infusion pump in pediatrics. Pediatr Allergy Immunol. 2013;24(1):49-53. doi:10.1111/pai.12026.

9. Cozon GJN, Clerson P, Dokhan A, Fardini Y, Sala TP, Crave JC. In-depth interviews of patients with primary immunodeficiency who have experienced pump and rapid push subcutaneous infusions of immunoglobulins reveal new insights on their preference and expectations. Patient Prefer Adherence. 2018;12:423-9. doi:10.2147/PPA.S156983.

10. Jolles S. Hyaluronidase facilitated subcutaneous immunoglobulin in primary immunodeficiency. Immunotargets Ther. 2013;2:125-33. doi:10.2147/ITT.S31136.

11. Jolles S, Orange JS, Gardulf A, Stein MR, Shapiro R, Borte M et al. Current treatment options with immunoglobulin G for the individualization of care in patients with primary immunodeficiency disease. Clin Exp Immunol. 2015;179(2):146-60. doi:10.1111/cei.12485.

12. Martin A, Lavoie L, Goetghebeur M, Schellenberg R. Economic benefits of subcutaneous rapid push versus intravenous immunoglobulin infusion therapy in adult patients with primary immune deficiency. Transfus Med. 2013;23(1):55-60. doi:10.1111/j.1365-3148.2012.01201.x.

13. Ponsford M, Carne E, Kingdon C, Joyce C, Price C, Williams C et al. Facilitated subcutaneous immunoglobulin (fSCIg) therapy--practical considerations. Clin Exp Immunol. 2015;182(3):302-13. doi:10.1111/cei.12694.

14. Schmidt RE, Ochs HD. 7th International Immunoglobulin Conference: Immunodeficiencies. Clin Exp Immunol. 2014;178 Suppl 1:21. doi:10.1111/cei.12497.

15. Wasserman RL, Melamed I, Stein MR, Gupta S, Puck J, Engl W et al. Recombinant human hyaluronidase-facilitated subcutaneous infusion of human immunoglobulins for primary immunodeficiency. J Allergy Clin Immunol. 2012;130(4):951-7 e11. doi:10.1016/j.jaci.2012.06.021.

16. van Schaik IN, Bril V, van Geloven N, Hartung HP, Lewis RA, Sobue G et al. Subcutaneous immunoglobulin for maintenance treatment in chronic inflammatory demyelinating polyneuropathy (PATH): a randomised, double-blind, placebo-controlled, phase 3 trial. Lancet Neurol. 2018;17(1):35-46. doi:10.1016/s1474-4422(17)30378-2.

17. Jolles S, Borte M, Nelson RP, Jr., Rojavin M, Bexon M, Lawo JP et al. Long-term efficacy, safety, and tolerability of Hizentra^®^ for treatment of primary immunodeficiency disease. Clin Immunol. 2014;150(2):161-9. doi:10.1016/j.clim.2013.10.008.

18. Kanegane H, Imai K, Yamada M, Takada H, Ariga T, Bexon M et al. Efficacy and safety of IgPro20, a subcutaneous immunoglobulin, in Japanese patients with primary immunodeficiency diseases. J Clin Immunol. 2014;34(2):204-11. doi:10.1007/s10875-013-9985-z.
